# Preliminary data on pupal development, lifespan and fertility of *Cynomya mortuorum* (L., 1761) in Belgium (Diptera: Calliphoridae)

**DOI:** 10.3897/BDJ.3.e5387

**Published:** 2015-07-31

**Authors:** Yves Braet, Luc Bourguignon, Sofie Vanpoucke, Valérie Drome, Françoise Hubrecht

**Affiliations:** ‡ Laboratoire Microtraces et Entomologie, Institut national de Criminalistique et de Criminologie, Chaussée de Vilvorde 100, B-1120, Brussels, Belgium

**Keywords:** Forensic science, forensic entomology, dimorphism, adult longevity, fertility

## Abstract

**Background:**

The calliphorid *Cynomya mortuorum* (L., 1761) is a species of forensic interest, present mainly in the Palaearctic Region. Nearly nothing is known about its life history.

**New information:**

We provide here the first data regarding pupal weight evolution during the pupal stage, female fertility and life expectancy of the species. At 22°C under a variable regime of temperatures, the egg-to-adult development time was an average of 18.05 ± 0.72 and 18.47 ± 0.67 days for females and males, respectively, in the control group. The pupal stage represented 56.7% of the total development. The development time from egg to adult and the duration of the pupal stage were significantly longer for males than for females. The measurement of pupal weight at the start of the pupal period revealed that female pupae were significantly lighter than male pupae by nearly 20%. This difference between the sexes was also observed for the dry weight of adults. An average decrease of 8.75% was observed throughout the first 8 days of the pupal stage, after which most adults started to emerge. The tested females produced an average of 176.13 ± 66.62 eggs throughout the egg-laying period. The average lifespan after emergence was 12.10 ± 4.09 days for females and 12.60 ± 2.95 days for males, with a median of 12.50 days for both sexes.

## Introduction

Blow flies (Diptera: Calliphoridae) have been important forensic indicators for over a century (Mégnin 1894, Leclercq 1974, Leclercq 1976, Lerclercq 1978, Wyss and Cherix 2006, Byrd and Castner 2010). Despite the increase of published data for several species, many remain understudied especially as to their biology, ethology and development. This is certainly the case for *Cynomya mortuorum* (L., 1761). This species is mainly Palaearctic but partly overlaps the distribution of *C. cadaverina* Robineau-Desvoidy, 1830 in Alaska and the Far East of Russia (Rognes 1991, Kurahashi and Kuranishi 2000). It is rarely abundant (Rognes 1991, Wyss and Cherix 2006, Frederickx et al. 2011), without a clear synanthropic preference (Nuorteva 1963), and is rarely encountered in forensic casework (Staerkeby 2001, Wyss and Cherix 2006). Few data have been obtained about its immature stages (described by Erzinçlioğlu 1985) or its development cycle (Nuorteva 1972, Nuorteva 1977 in Rognes 1991). Rognes 1991 reported that it can be found in animal burrows and that it has been reared from small mammal carcasses. It was also recorded as an agent of myiasis on a hare. Recently, the thermal constant of this species was published (Braet et al. 2015). Since *C. mortuorum* was found in one forensic case in Belgium, we decided to obtain more data on the life-traits of this species.

## Material and methods

### Origin and rearing of adults

Specimens for the laboratory colonies of *Cynomya mortuorum* used in this study were collected in Outrelouxhe, Belgium (altitude 250m), on May 1^st^, 2012. The adult fly colonies were maintained in mesh rearing cages (40cm x 40cm x 40cm) at room temperature (15° and 21°C during night and day, respectively), with a photoperiod of 16:8 (L:D; hours). Each cage contained 150–200 flies. They were provided *ad libitum* with water, sugar, and powdered milk. After emergence and over a period of three days, the adult flies were provided with a piece of beef heart as a protein source to promote the ovarian development of females (Berkebile et al. 2006). The original specimens were identified with the identification key provided by Dr K. Szpila for EAFE members, and compared to the specimens housed in the "Institut Royal des Sciences Naturelles de Belgique". Voucher specimens were preserved in the collection of the Belgian National Forensic Institute.

### Rearing conditions of larvae and pupae

When eggs were required, a dish containing one piece of beef heart (around 10g) was placed into one or several mesh cages and removed 4 hours later. Each piece of meat with the newly laid eggs was transferred (for a maximum of 250–300 eggs) onto approximately 250g of fresh minced beef heart. Each portion of fresh minced beef heart was finally placed into home-made plastic rearing boxes (23cm x 17.5cm x 10cm). A 1cm thick layer of dry sand covered the bottom of the boxes to provide a pupation medium. The rearing boxes were then placed in an environmental chamber (SANYO Incubator MIR 553, Sanyo Electric Biomedical, Japan). To ensure a suitable relative humidity (> 85%), a container filled with water was also placed inside the environmental chamber.

The rearing treatments were maintained at a photoperiod of 16:8 (L:D; hours) and at a temperature gradually changing (over a 15 minute period) ​from 18°C to 24°C between the dark and light periods. This resulted in an average temperature of 22°C. A data logger (Testo 175-T1) was inserted into the incubator to monitor the temperature hourly.

### Pupal weight evolution before emergence

From the eggs and larvae reared in the conditions described above, all the pupae were sampled for our analyses. The one-day old pupae (light brown in colour) were placed in 24-well plates (one pupa in each well), which were then placed back in the environmental chamber. All plates were divided into two batches. Weighing of the pupae began the day after the sampling and was undertaken on a Mettler-Toledo AB104-S balance (± 0.1 mg). The pupae of the first batch (Control group) were weighed only once and then replaced and left undisturbed in the incubator until the emergence of the adults. The pupae of the second batch (Test group) were weighed each day until the emergence of the adults, and immediately placed back in their individual well and in the incubator between two measurements. Successfully emerging adults were stunned with a small amount of CO_2_ before being removed from the rearing box. To avoid temperature bias, the rearing boxes were randomly replaced inside the incubator.

At emergence, the developmental time was recorded. The adults were sexed and dried for 15 days (at 40°C, RH: 0%) and then weighed. The change in pupal weight was recorded only during the first 8 days of the pupal stage. The last two days were not taken into account as the remnant number of pupae to emerge was very low.

### Estimation of fertility

Ten newly emerged couples were isolated in small plastic rearing boxes (12cm x 9cm x 5cm) and provided *ad libitum* with sugar and water. The couples were maintained at an average temperature of 22°C: 24°C and 18°C during day and night, respectively (16:8 L:D photoperiod), with a 15 min changeover period between the two temperatures. One piece of fresh beef heart (around 10g) was provided every day. Each day at 10:00AM, when replacing the piece of meat with a new one, a short puff (of maximum 2 seconds) of CO_2_ was used to keep the flies calm. As the flies could lay over several days or just one day, the removed piece of meat was examined for the presence of eggs. The death of specimens and the number of laying events, eggs and larvae were also recorded daily.

### Data analysis

Statistical analysis was performed using Microsoft® Excel 2010/XLSTAT^©^-Pro (Version 2013.5.08, Addinsoft, Inc., Brooklyn, NY, USA), the significance level was set at *P*≤0.05. Equality of variances between each set was confirmed with an *F*-test.

## Results

### Pupal weight change during development

The total number of pupae collected was 381 (Control: 189; Test: 192). The overall percentage of emergence was 81.89% (Control: 86.24%; Test: 77.60%). After elimination of the non-emerged pupae, as well as dead pupae or pupae presenting deformities, a total number of 309 pupae were studied and tested (Control: 161; Test: 148).

Among the retained pupae, the observed sex ratio was 1.70 males per female in the Control group and 1.59 males per female in the Test group. Maximum egg-to-adult development time was 20 days for the Control group with an average of 18.21 ± 0.73 days (18.05 ± 0.72 and 18.47 ± 0.67 days for females and males, respectively). For the Test group, the maximum egg-to-adult development time was 21 days with an average of 18.39 ± 0.91 days (18.23 ± 0.90 and 18.65 ± 0.89 days for females and males, respectively) (Table 1). The average duration of the pupal stage represented 56.7% of the total development period in the Control group and 56.6% in the Test group. The differences observed between batches for each sex were not significant.

Within each batch, the development time from egg to adult was significantly longer for males than for females (Table 1) (t test, ≤ 0.008). As for the duration of the pupal stage and ADD observed (Braet et al. 2015), the observation was the same.

The average pupal weight measured one day after sampling was 103.87 ± 14.66mg (95.2 ± 9.49mg for females, 117.72 ± 10.08mg for males) in the Control group and 101.51 ± 15.16mg (93.4 ± 9.29mg for females, 115.62 ± 12.9mg for males) for the Test group. The observed differences were not significant. In both groups, the female pupae were significantly lighter (80.87% of the weight of male pupae in the Control group, 80.78% in the Test group) (t test, <0.0001). This was also observed for the dry weight of adults, on average equal to 17.33mg for the Control group and 22.79mg for the Test group. In both batches, adult female flies presented a significantly lower dry weight than males (77.18% of the weight of adult males in the control group, 81.55% in the test group) (t test, <0.0001) (Table 1).

Between the first day and the eighth day of weighing, an average decrease of 8.75% was observed in the Test group (8.40% for female pupae; 9.26% for male pupae) (Fig. 1).

### Estimation of fertility and lifespan

**Figure 2.**
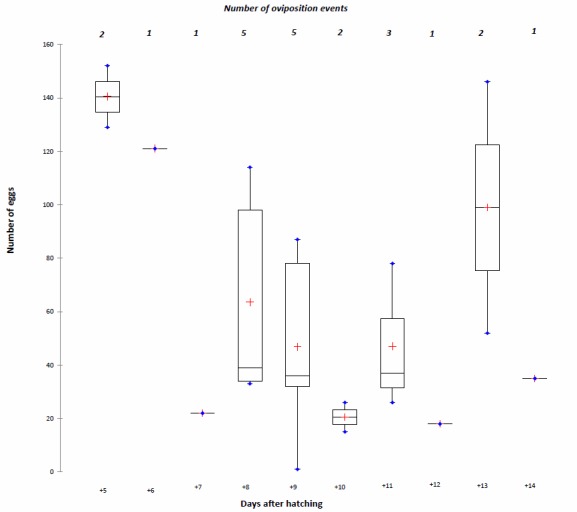
Number of oviposition events and of eggs laid after hatching of pupae. The red cross represents the median. (Suppl. material 2)

Ten pairs of *C. mortuorum* laid a total of 1,409 eggs during their life. The egg-laying period extended from 5 days after emergence for early emerging couples to 14 days after emergence for the last emerging couple (Fig. 2). The number of egg-laying events reached its maximum between 8 and 9 days after emergence (5 egg-layings observed). The number of eggs laid regularly decreased during the period (Fig. 2), with an average of 140.5 eggs 5 days after emergence (Fig. 2). Each pair produced an average of 176.13 ± 66.62 eggs throughout the laying period (Table 2).

The average lifespan after emergence was 12.10 ± 4.09 days for females and 12.60 ± 2.95 days for males (Table 2). Lifetime medians were the same for both sexes, i.e. 12.50 days.

## Discussion

*Cynomya mortuorum* (Diptera, Calliphoridae) is a large, conspicuous species but is not frequently collected by entomologists (Frederickx et al. 2011). This species appears in only one forensic case in Belgium. When reared, it is a calm species that is less easily disturbed than other Calliphoridae, such as *Lucilia sericata* (Meigen, 1826), and the adult flies show a weak attraction to UV (unlike *Lucilia sericata* and *Phormia regina* (Meigen, 1826))(unpublished observations).

### Pupal weight change during development

The lack of a significant difference between the Control group and the Test group indicates that the daily weighing of pupae in the test batch did not affect pupal development.

The observed sex ratio was an average of 1.65 males for every female. There were no significant differences between the Control group and the Test group regarding development time (egg-to-adult or pupa-to-adult). At 22°C, the pupal stage represented just over half of the total immature development period. The observed duration of the pupal stage of *C. mortuorum* (10.32 ± 0.53 days in the Control group and 10.41 ± 0.6 days in the Test group) was between the values estimated by Marchenko 2001) for *C. vicina* and *C. vomitoria*, two other members of the tribe Calliphorini (respectively 9.8 days and 13.6 days). Similarly, there were no significant differences between groups regarding the average weight of pupae or the average dry weight of adults.

During our tests, it appears that males of*C. mortuorum* have a slightly longer developmental time than females. Both pupal weight and dry weight of adult males were about 1.2 times higher than females. During the first 8 days of the pupal stage, the male pupae exhibited a higher loss in mass than female pupae (9.26% vs. 8.40%, respectively). To our knowledge, this decrease in mass during the pupal stage has never been reported in studies on the development of Diptera of forensic importance. However, this observation was not completely unexpected, given that a loss in water content is inevitable and a mechanical consequence of respiration (Chown et al. 2011, Rivers et al. 2013). Furthermore, a decrease in the mass could also indicate a reduction in the fatty masses, which are hydrolyzed to provide energy during the pupal stage as shown for *Lucilia sericata* (Muntzer et al. 2015​).

The significant difference in mass between male and female *C. mortuorum* was also confirmed in the adult size (data not shown). This sexual size dimorphism (SDS) may reflect the existence of a phenomenon of sexual competition between males of different sizes as observed in certain strains of *Drosophila melanogaster* Meigen and many Chironomidae (Diptera) (Partridge and Farquhar 1983). Another hypothesis is that a differential in the use of resources could reduce competition between genders and lead each gender to use different niches (Selander 1966, Shine 1989, Sandercock 2001, Temeles and Kress 2003), though this hypothesis has not been verified in insects.

### Estimation of fertility and lifespan

Despite the greater mass of the males, their longevity was barely higher than that of females (12.6 days vs. 12.1 days, respectively). This is contrary to what is observed in some other fly species (Partridge and Farquhar 1983, Neems et al. 1990, Abou Zied et al. 2003), where females live longer. This could be related to the presence of relatively large and strongly sclerotized genital structures compared to males of other blowfly species, that could have a negative impact on the life expectancy of males. Under the conditions of our tests, the first oviposition of *C. mortuorum* was observed 5 days after emergence of the adults. All the egg-laying took place during the next 9 days with a maximum observed 8–9 days after emergence. Each female laid an average of 173.38 ± 63.56 eggs throughout the oviposition period. This amount is rather low in comparison to other Calliphoridae. For exemple, the average lifetime fecundity of *Lucilia cuprina* ​(Wiedemann, 1830) is between 232 and 445.69 eggs per female (Vogt et al. 1985, Abou Zied et al. 2003), while *Cochliomyia hominivorax* (Coquerel, 1858) and *Chrysomya bezziana* (Villeneuve, 1914) can lay 200–400 and 190–250 per batch, respectively (Spradbery 2002). Together, the adult longevity and the rather low amount of eggs laid during the life cycle suggest more investigation is needed to verify if *C. mortuorum* follows an r- or a K-strategy.

These new data, while preliminary, bring some information on the development of *Cynomya mortuorum*. More work is needed, however, especially to understand the low frequency of collection of this species.

## Supplementary Material

Supplementary material 1 Raw Data for Fig.1a & 1b Data type: Datasheet
Brief description: This file contains the values of the evolution of pupal weight (mg) for males and females during the 8 days after sampling in the Test group experiment. The initial value of the pupal weight in the Control group is also shown for comparison with the initial value of the pupal weight in the Test group.
File: oo_44311.xls Braet Y., Bourguignon L., Vanpoucke S., Drome V. & Hubrecht F.

Supplementary material 2 Raw Data for Fig 2 Data type: Datasheet
Brief description: Number of oviposition events and of eggs laid after hatching of the pupae. The red cross represents the median.
File: oo_47177.xls Braet Y., Bourguignon L., Vanpoucke S., Drome V. & Hubrecht F.

## Figures and Tables

**Figure 1a.**
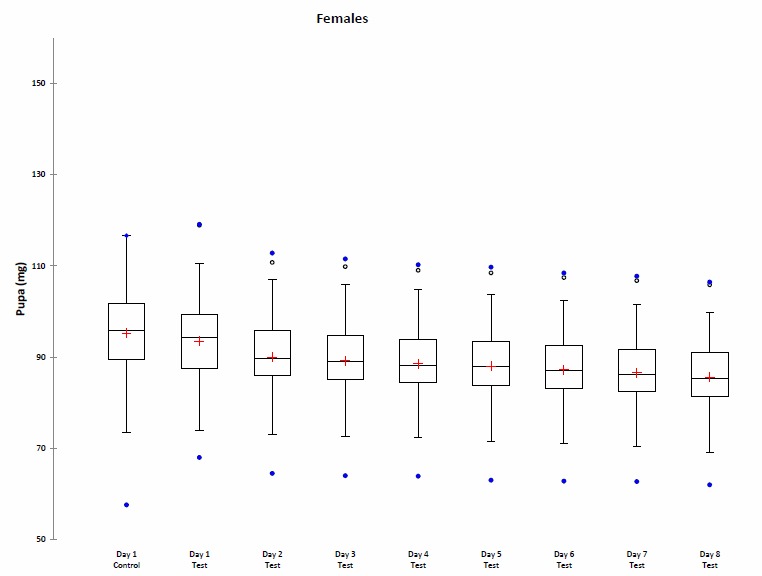


**Figure 1b.**
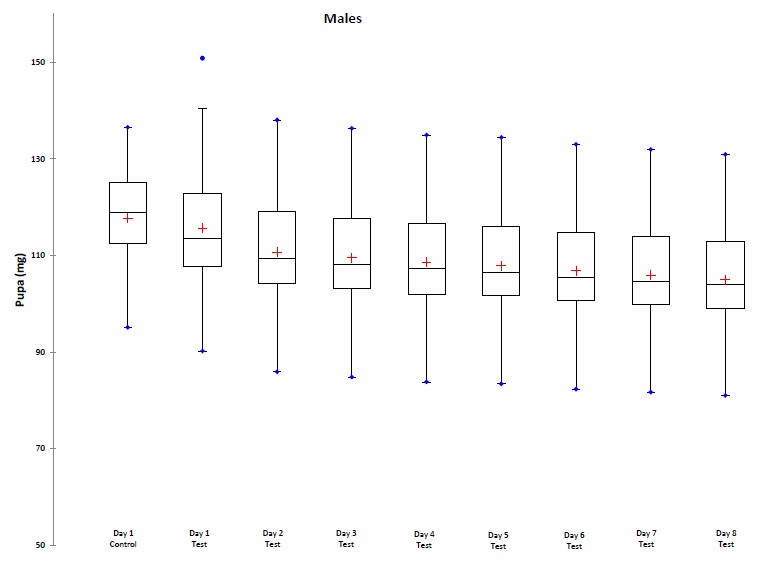


**Table 1.** Mass, developmental time (in days) and accumulated degree days (ADD) between the Control and the Test (daily weighing) for the retained pupae. (SD) = Standard deviation in brackets for each value. *: measure of the pupa was done one day after sampling. ^$^: ADD was calculated for the egg–adult development time at 22°C using the lower threshold given by Braet et al. 2015).

**Mean (SD)**		Pupae weight (mg)*	**Adult dry weight (mg)**	**Developmental time (d)**		**ADD^$^(°C days)**
				**Egg - Adult**	**Pupa - Adult**	
**Control**	**All**	103.87 (14.66)	17.33 (3.26)	18.21 (0.73)	10.32 (0.53)	292.18 (12.68)
	**Female**	95.20 (9.49)	15.56 (2.12)	18.05 (0.72)	10.22 (0.53)	289.39 (12.53)
	**Male**	117.72 (10.08)	20.16 (2.73)	18.47 (0.67)	10.47 (0.50)	296.65 (11.68)
**Test**	**All**	101.51 (15.16)	22.79 (3.31)	18.39 (0.91)	10.41 (0.60)	295.21 (15.93)
	**Female**	93.40 (9.29)	21.05 (2.07)	18.23 (0.90)	10.35 (0.56)	292.58 (15.62)
	**Male**	115.62 (12.90)	25.81 (2.88)	18.65 (0.89)	10.50 (0.67)	299.79 (15.55)

**Table 2.** Lifespan (in days) of males and females for each rearing box with the corresponding amount of eggs laid by the female during its life. *: zero scores were not used in calculating the average, SD and median.

**Couple number**	**Lifespan (d)**		**Number of eggs**
	**Female**	**Male**	
**01**	17	14	139
**02**	18	18	263
**03**	13	13	196
**04**	10	10	187
**05**	7	9	121
**06**	12	15	62
**07**	15	15	192
**08**	13	12	0*
**09**	11	11	249
**10**	5	9	0*
**Mean (SD)**	12.10 (4.09)	12.60 (2.95)	176.13 (66.62)
**Median**	12.50	12.50	189.50
